# BJS and BJS Open correspondence to move to the BJS Academy

**DOI:** 10.1093/bjsopen/zrae077

**Published:** 2024-07-02

**Authors:** Ville Sallinen, Desmond C Winter, Jonothan J Earnshaw

**Affiliations:** *BJS Open Editor-in-Chief*; *BJS Editor-in-Chief*; *BJS Academy Director*

The two BJS journals include correspondence referring to the scientific articles they publish. The Academy has a discussion area—Cutting Edge Blog—that contains short commentaries on articles published in BJS and BJS Open, to add value to the publications. The new idea is to merge correspondence and comment in a single place to maximize the opportunity for readers to engage with sources to discuss and critique the published research. The aim is to have a single area with all the comment and discussion on articles from BJS journals (and all surgical matters) that readers will visit to keep up to date with surgical debate and discussion.

From 14 July 2024, all correspondence to BJS, BJS Open and BJS Academy will be submitted through a single area: correspondence@BJSAcademy.com. Submissions will be processed in the normal way by journal editors and the Academy team, with the aim to publish accepted material no more than 14 days after submission. The usual Author Guidelines apply, which can be found at https://academic.oup.com/bjsopen/pages/general-instructions (section h under the heading Article Types). In particular, the Academy will be mindful of plagiarism and the intentional use of artificial intelligence (AI) to create material. At present, BJS Academy will not accept AI as a co-author of correspondence.

In the coming months, the aim is to expand content in the BJS Academy Cutting Edge Blog to make it the go-to place for surgeons to read opinion and controversy about BJS and BJS Open material, and to create a vibrant and interesting area for discourse and discussion.

